# Light Weight Bedding

**Published:** 1891-03

**Authors:** 


### LIGHT WEIGHT BEDDING.

All bed coverings ought to be as light as is consistent with warmth, and therefore woolen blankets are far more healthful than the ordinary heavy comforter, which admits of no ventilation, but absorbs and retains the exhalations from the body. Blankets can be washed often, while comforters cannot, unless taken entirely to pieces. In regard to the airing of the bedding and sleeping room, one cannot be too particular. Choosing bright, sunny days, mattresses, pillows, and each separate article of bedding should be turned out of door several times each week.

